# Carbamoylation correlates of cyanate neuropathy and cyanide poisoning: relevance to the biomarkers of cassava cyanogenesis and motor system toxicity

**DOI:** 10.1186/2193-1801-2-647

**Published:** 2013-12-02

**Authors:** Samuel Kimani, Victor Moterroso, Mike Lasarev, Sinei Kipruto, Fred Bukachi, Charles Maitai, Larry David, Desire Tshala-Katumbay

**Affiliations:** Department of Pharmacology and Pharmacognosy, University of Nairobi, Nairobi, 19676 Kenya; Department of Comparative Medicine, Oregon Health & Science University (OHSU), Portland, OR 97239 USA; Center for Research on Occupational & Environmental Toxicology, OHSU, Portland, OR 97239 USA; School of Nursing Sciences, University of Nairobi, Nairobi, 19676 Kenya; Department of Medical Physiology, University of Nairobi, Nairobi, 30197 Kenya; Biochemistry and Molecular Biology & Proteomics Shared Resource, OHSU, Portland, OR 97239 USA; Department of Neurology, OHSU, Portland, OR 97239 USA; Center for Research on Occupational and Environmental Toxicology & Department of Neurology, Oregon Health & Science University, 3181 Sam Jackson Park Road, Mail code L606, Portland, OR 97239 USA

**Keywords:** Carbamoylation, Cyanate, Cyanide, Neuropathy, Proteomics

## Abstract

We sought to elucidate the protein carbamoylation patterns associated with cyanate neuropathy relative to cyanide poisoning. We hypothesized that under a diet deficient in sulfur amino acids (SAA), the carbamoylation pattern associated with cyanide poisoning is similar to that of cyanate neuropathy. Male rats (6–8 weeks old) were fed a diet with all amino acids (AAA) or 75%-deficiency in SAA and treated with 2.5 mg/kg/body weight (bw) NaCN, or 50 mg/kg/bw NaOCN, or 1 μl/g/bw saline, for up to 6 weeks. Albumin and spinal cord proteins were analyzed using liquid chromatography mass spectrometry (LC-MS/MS). Only NaOCN induced motor deficits with significant levels of carbamoylation. At Day 14, we found a diet-treatment interaction effect on albumin carbamoylation (p = 0.07). At Day 28, no effect was attributed to diet (p = 0.71). Mean number of NaCN-carbamoylated sites on albumin was 47.4% higher relative to vehicle (95% CI:16.7-86.4%). Only NaOCN carbamoylated spinal cord proteins, prominently, under SAA-restricted diet. Proteins targets included myelin basic and proteolipid proteins, neurofilament light and glial fibrillary acidic proteins, and 2', 3' cyclic-nucleotide 3'-phosphodiesterase. Under SAA deficiency, chronic but not acute cyanide toxicity may share biomarkers and pathogenetic similarities with cyanate neuropathy. Prevention of carbamoylation may protect against the neuropathic effects of cyanate.

## Background

Dietary dependency on cyanogenic cassava and low intake in sulfur amino acid (SAA) have been implicated in outbreaks of a central motor neuron disease known as konzo. Subjects affected by the disease present with an overt spastic abnormality of their gait or a complete inability to walk (Howlett et al. 1990Tylleskar et al. 1992Tylleskar et al. 1994Chabwine et al. 2011Cliff et al. 2011Boivin et al. 2013). We recently showed that children from konzo-affected areas may also present with a pervasive cognitive dysfunction suggesting that the clinical burden associated with cassava cyanogenic intoxication has been underestimated (Boivin et al. 2013). The exact biomarkers and mechanisms of the disease remain unclear. The disease mainly affects children and women of childbearing age among sub-Saharan African populations that subsist on food products derived from insufficiently processed bitter and cyanogenic cassava.

Cassava is a carbohydrate-enriched and cyanogenic crop with a very low content in proteins and only 1-2% content in SAA, which are needed for humans to detoxify cyanide (Diasolua Ngudi et al. 2002Kassa et al. 2011Nunn et al. 2011). It contains linamarin, a cyanogenic compound that is metabolically converted to cyanohydrins and hydrogen cyanide, a well-known mitochondrial toxin. Under normal conditions, cyanide (CN) is converted to a less toxic compound i.e. thiocyanate (SCN) via the SAA-dependent rhodanese pathway, to trace amounts of cyanate (OCN), and to 2-iminothiazoline-4-carboxylic acid. Under conditions of SAA-deficiency, oxidative detoxification pathways of cyanide are favored and there is increased production of OCN, a well-known motor system toxin (Tellez et al. 1979Swenne et al. 1996Spencer 1999Tor-Agbidye et al. 1999aZottola et al. 2009). Epidemiological studies have shown a link between cassava cyanogenic exposure, protein malnutrition, and outbreaks of konzo (Banea-Mayambu et al. 1997Cliff et al. 2011Nzwalo and Cliff 2011). Currently, research efforts focus on elucidating the biomarkers and mechanisms of the disease. Limited progress has been made partially due to the lack of experimental models.

We previously showed that proteomics methodologies may help unveil biomarkers of cyanogenic exposure (Kassa et al. 2011). In this study, we compared the carbamoylation patterns of cyanate neuropathy versus cyanide poisoning under a diet deficient in SAA. We hypothesized that under a diet deficient in sulfur amino acids (SAA), the carbamoylation pattern associated with cyanide poisoning is similar to that of cyanate neuropathy. Liquid chromatography tandem mass spectrometry (LC-MS/MS) analysis was performed on albumin and spinal cord proteins from rodents treated with cyanide or cyanate while fed with either a normal (all amino acid-containing) diet or a diet deficient in SAA. NaOCN, or NaCN to a lesser extent, carbamoylates serum albumin. SAA deficiency exacerbates the NaOCN carbamoylation effects on select spinal cord proteins. Studies on humans affected by cassava-associated neurological diseases are needed to determine whether chronic cassava cyanogenic exposure is associated with cyanate-like carbamoylation patterns.

## Results

### Motor deficits

**Figure 1:**
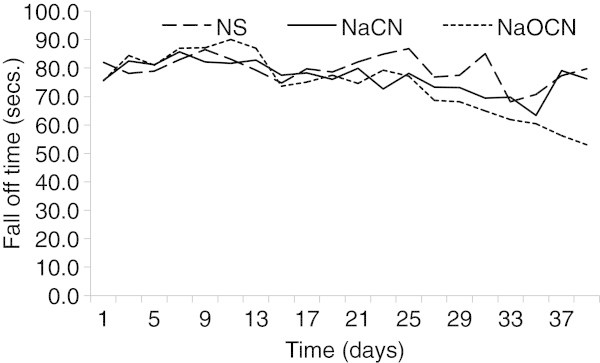
**Rotarod performance in rats treated with normal saline (NS), NaCN or NaOCN and fed SAA-restricted diet.** Treatment affected rotarod fall off time in rats. The fall time from the rotating rod decreased significantly (p <0.001) among rats treated with NaOCN compared to control and NaCN overtime.

Over the course of the study, the odds of remaining on the rotarod for at least 60s was significantly (p < 0.05) lower for the SAA-fed rats treated with NaOCN compared to those treated with NaCN or vehicle. Treatment affected the rotarod fall off time. The fall time was also significantly (p = 0.040) shorter on the last testing day in animals treated with NaOCN compared to control and NaCN groups. No statistical difference was found between NaCN relative to the control treated groups (p = 0.16) (Figure 1).

### Albumin carbamoylation

There was a significant interaction between diet and treatment with respect to mean number of assigned MS/MS spectra to carbamoylated albumin peptides (spectral counts) at 14-day time point (X^2(2df) = 5.24, p = 0.073). NaOCN overtly induced high levels of carbamoylation of albumin relative to vehicle or NaCN regardless of the diet (P < 0.001) (Table 1).

**Table 1:** **Day 14 mean number of spectral counts (95% CI mean response) for carbamoylated albumin peptides**

	Diet	
Treatment	AAA estimates (95%CI)	SAA estimates (95%CI)
Vehicle	22.5 (16.5 - 30.7)	15.8 (11.2 - 22.1)
NaCN	18.0 (13.0 - 25.0)	21.5 (15.7 - 29.4)
NaOCN	521.0 (411.0 - 660.0)	702.0 (554.0 - 888.0)
**Comparisons**	**Estimated ratios (95% CI)**	**Estimated ratios (95% CI)**
NaCN/Vehicle	0.80 (0.51 - 1.26)	1.37 (0.86 - 2.17)]
NaOCN/Vehicle	23.2 (15.7 - 34.2)*	44.5 (29.5 - 67.3)*
NaOCN/NaCN	28.9 (19.3 - 43.4)*	32.6 (22.0 - 48.3)*

NaOCN overtly (*) induced high levels of carbamoylation of albumin relative to vehicle or NaCN regardless of the diet.

At the 28-day time point, there was no evidence of interaction between diet and treatment with respect to mean number of spectral counts for carbamoylated albumin peptides (X^2(2df) = 3.83, p = 0.15), thereby simplifying comparisons across treatments and diets. The mean number of spectral counts for albumin carbamoylated peptides was 47.4% (95% CI: 16.7 - 86.4%) greater in NaCN-treated animals relative to their vehicle-treated counterparts (p = 0.001), regardless of diet. The carbamoylation effect was amplified in animals treated with NaOCN compared to vehicle, with the mean response for NaOCN animals being 33.9 (95% CI: 28.0 - 41.1) times as much as the mean response for vehicle (p < 0.001) or 23 (95% CI: 19.5 - 27.2) times the mean response for NaCN (p < 0.001) (Table 2). After controlling for treatment, we found no significant differences in carbamoylation that could be attributed to diet (p = 0.71).

**Table 2:** **Day 28 mean number of spectral counts (95% CI mean response) for carbamoylated albumin peptides**

	Diet	
Treatment	AAA estimates (95% CI)	SAA estimates (95% CI)
Vehicle	17.3 (14.3 - 20.9)	16.9 (14.0 - 20.5)
NaCN	25.5 (21.7 - 30.0)	25.0 (21.2 - 29.4)
NaOCN	587.0 (535.0 - 644.0)	574.0 (525.0 - 629.0)

Carbamoylation effects were significantly seen in NaCN and NaOCN-treated animals relative to their vehicle-treated counterparts, with a pronounced effect of NaOCN regardless of the diet.

When comparing the levels of albumin carbamoylation at Day 14 vs. Day 28, we found no evidence of an interaction between diet and treatment (X2(2df) = 2.27, p = 0.32). With the exception of animals treated with NaCN under the normal AAA diet, the relative change in levels of carbamoylation was not significant. In the NaCN/AAA group, the mean number of spectral counts for carbamoylated albumin peptides was estimated to increase 55% (95% CI: 20 - 101%; p = 0.001) over the 14-day span. All other entries, however, showed relative changes not significantly different (p ≥ 0.15) from unity (Table 3).

**Table 3:** **Day 28/Day14 ratios (95% CI) of mean numbers of spectral counts for carbamoylated albumin peptides**

	Diet	
Treatment	AAA ratios (95% CI)	SAA ratios (95% CI)
Vehicle	1.06 (0.80 - 1.40)	0.80 (0.61 - 1.07)
NaCN	1.55 (1.20 - 2.01)*	1.18 (0.91 - 1.53)
NaOCN	1.17 (0.94 - 1.46)	0.89 (0.71 - 1.11)

Comparison of the levels of albumin carbamoylation at day 28 vs. day 14 revealed increased carbamoylation (*) in the group of animals treated with NaCN and fed with a normal diet.

### Spinal cord proteomics

On the last experimental day, we found a differential pattern of susceptibility to carbamoylation in spinal cord proteins. Carbamoylation was almost exclusively induced by NaOCN so that comparisons between diets were limited to this one neurotoxicant. NaOCN-targeted proteins included myelin basic protein (MBP), myelin proteolipid protein (MPP), neurofilament light polypeptide (NFLP), glial fibrillary acidic protein (GFAP), and 2', 3' cyclic-nucleotide 3'-phosphodiesterase. MBP was 18.8% (95% CI: 2.3 - 38.1%; p = 0.024) more carbamoylated in animals under the SAA-restricted diet relative their AAA counterparts. With regard to MPP, animals in the SAA-restricted diet had 23.3% (95% CI: 5.8 - 43.7%; p = 0.007) more carbamoylation sites compared to their AAA counterparts. Similarly for NFLP, the SAA-restricted diet had an impact on carbamoylation. Under the SAA-restricted diet, the animals had 39% (95% CI: 2.6 - 88.2%; p = 0.03) more carbamoylation sites relative to their AAA counterparts. For GFAP, the SAA-deficient group had 34.2% (95% CI: 5.9 - 69.9%; p = 0.02) more carbamoylation sites relative to the AAA-group. With regard to 2', 3' cyclic-nucleotide 3'-phosphodiesterase, the SAA-restricted diet produced an average number of carbamoylation sites that was 2.85 (95% CI: 1.8 - 4.5; p < 0.001) times the corresponding average response of animals fed the normal AAA diet (Table 4).

**Table 4:** **NaOCN-induced mean numbers of carbamoylated sites (95% CI the mean response) on spinal cord proteins**

Protein	AAA Diet	75% SAA-Deficient diet
Myelin basic protein	78.25 (70.0 - 87.4)	93 (84.0 – 102.0)
Myelin proteolipid protein	74 (66.0 – 82.9)	91.25 (82.4 – 101.0)
Neurofilament light polypeptide	48.75 (39.0 – 60.8)	67.75 (55.0 - 83.4)
Glial fibrillary acidic protein	30 (25.1 - 35.9)	40.25 (34.5 - 47.0)
2', 3' cyclic-nucleotide 3'-phosphodiesterase	6.5 (4.4 – 9.6)	18.5 (14.6 – 23.5)

Carbamoylation pattern in spinal cord proteins indicates an exacerbating effect of the SAA-deficient diet, mostly for 2', 3' cyclic-nucleotide 3'-phosphodiesterase.

None of the animals in the Vehicle- or NaCN-treated groups (either diet) had any carbamoylated sites for MBP or GFAP. For MPP, only one animal had 1 carbamoylated site in the Vehicle/AAA group; all other Vehicle and NaCN treatments (both diets) were otherwise uniformly silent. For the NFLP, only one animal had 1 carbamoylated site in the NaCN/SAA group; all other Vehicle and NaCN treatments (both diets) were otherwise uniformly silent.

## Discussion

We used state-of-the art proteomic methodologies to reveal differential carbamoylation patterns associated with cyanate neuropathy versus cyanide poisoning under conditions of dietary deficiencies in SAA. Results indicate that carbamoylated albumin may be a useful marker of exposure to cyanate and carbamoylating derivatives or precursors such as urea. Only NaOCN induced motor deficits and significant carbamoylation of albumin and select spinal cord proteins. On the assumption that these findings would hold true for additional experimental time points and time dependent correlations between levels of carbamoylation and NaOCN-induced deficits in a large sample of rats, our results suggest that cyanate neuropathy may be mediated through carbamoylation. This proposal is consistent with earlier studies that have shown a positive correlation between memory deficits and levels of carbamoylation in rat brain (Crist et al. 1973) or suggested that carbamoylated albumin may be clinically used to predict risks for neuropathy in uremic patients (Kraus and Kraus 2001Berg et al. 2013Kalim et al. 2013). The observed pattern of carbamoylation is consistent with the myelinotoxic effects of cyanate as previously reported (Ohnishi et al. 1975Tellez-Nagel et al. 1977Tellez et al. 1979). NaOCN targets include major myelin proteins and corresponding binding proteins such as neurofilament light polypeptide, glial fibrillary acidic protein, and 2', 3' cyclic-nucleotide 3'-phosphodiesterase; with the latter showing a greater carbamoylation effect. The impact of NaOCN on 2', 3' cyclic-nucleotide 3'-phosphodiesterase and, hence, functions of 2',3'-cyclic nucleotides to 2'-nucleotides in relation to microtubules and cytoskeleton organization, and cell survival, needs further attention. Establishing a model of cytoskeletal disorganization in response to cyanogenic poisoning may be relevant for testing of drug candidates including the neuroprotective peptide NAP (Zemlyak et al. 2009). The aforementioned pattern of protein susceptibility to carbamoylation should inform such studies to elucidate the exact pathways and mechanisms leading to cyanate neuropathy (Han et al. 2013).

The exacerbating effect of dietary SAA-deficiency on the carbamoylation of the aforementioned proteins has yet to be explained. In serum, SAA deficiency had no impact on the carbamoylation of albumin after 28-days of treatment with cyanate. This may possibly be explained by a “sulfur buffering effect” by abundant serum sulfur donors including albumin (Westley et al. 1983). Lack of increase in cyanate carbamoylation on day 28 relative to day 14 may be due to a metabolic clearance of carbamoylated proteins. For cyanide, however, the increase in levels of carbamoylation could simply portray a cumulative effect of cyanide detoxification with cyanate production and subsequent carbamoylation of albumin. This proposal would need, however, to be confirmed with quantitative measures of tissue contents in cyanide, cyanate, and SAA; which, along with a small sample size, are among the limitations of this study.

Within our study time frame, we failed to confirm the primary hypothesis that under SAA deficiency, acute cyanide toxicity induces carbamoylation patterns similar to that of cyanate neuropathy. However, in light of the observed response on albumin, chronic exposure to cyanide may conceivably lead to greater carbamoylation effects. This hypothesis should be tested on subjects chronically exposed to cyanide notably those living in konzo-affected areas. In these undernourished subjects, cyanide is hypothesized to undergo oxidative detoxification with subsequent increase in the production of cyanate, a well-known carbamoylating neurotoxin. The neurotoxic insults seen in these subjects could arise from a “multiple hit” process that combines of a direct mitochondrial hit by cyanide, a protein hit through carbamoylation, and a thiol-redox hit due to SAA-deficiency (Tor-Agbidye et al. 1999aTor-Agbidye et al. 1999bKassa et al. 2011Berg et al. 2013). Since only NaOCN-treated animals developed motor toxicity and carbamoylation of spinal cord proteins, our findings are in favor of a recent proposal that suggests that blocking carbamoylation may have neuroprotective effects in uremic subjects (Berg et al. 2013).

## Conclusion

The carbamoylation and neurotoxicity effects of cyanate may be influenced by diet with an exacerbating effect of dietary deficiency in sulfur amino acids. Cyanate hits proteins that are important in maintaining the shape and organization of the cytoskeleton, and hence, supporting mechanisms of neuronal survival. Proteins targets included myelin basic and proteolipid proteins, neurofilament light and glial fibrillary acidic proteins, and 2', 3' cyclic-nucleotide 3'-phosphodiesterase. Under SAA deficiency, chronic but not acute cyanide toxicity may share biomarkers and pathogenetic similarities with cyanate neuropathy. Prevention of carbamoylation may protect against the neuropathic effects of cyanate. Studies on the biomarkers and mechanisms of food (cassava) associated neurological diseases should consider the potential role of cyanate and its carbamoylation effects as endpoints of interest both at the experimental, clinical, and public health standpoints.

## Materials and methods

### Chemicals

Sodium cyanide (NaCN CAS No. 143-33-9, 97.2% purity) and cyanate (NaOCN CAS No. 917-61-3, 96% purity) were bought from Sigma–Aldrich (St. Louis, MO) and stored at room temperature. All other laboratory reagents were of analytical or molecular biology grades.

### Animals

Young adult male heterozygous rats (Crl: NIH-Fox1 rnu/Fox 1+, 6–8 weeks old) (N = 52), weighing 140-210 g upon arrival were donated by Professor Neuwelt, Department of Neurology, Oregon Health & Science University (OHSU). They were caged in an animal room maintained on a 12/12 h light/dark cycle in the Center for Research on Occupational and Environmental Toxicology (CROET). Food and water were given *ad libitum*. Experimental protocols were approved by the OHSU institutional animal care and use committee (IACUC).

### Diet and dosing regimens

#### Diet

Custom-synthesized isonitrogenous rodent diet with either all amino acids (AAA-diet; code TD09460) or lacking 75% of the SAA-content relative to the control diet (SAA-deficient diet; code TD09463) were purchased from Harlan (Madison, WI) and stored at 4°C until use. We chose a 75%-deficient but not free SAA to allow for animal survival and cyanide detoxification since previous experimentation showed animals fed with SAA-free chow had dramatic weight loss and muscle weakness (Tor-Agbidye et al. 1999a).

#### Dosing regimens

Animals were first acclimated for a 5-day period on a diet consisting of 4:1 portions of normal rodent chow (PMI Nutrition International, NJ) with either AAA-diet (N = 28) or 75%-SAA deficient diet (N = 24). On the 6^th^ day, they were assigned to experimental groups (N = 7-10/group) and treated intraperitoneally (one injection per day) for up to 6 weeks as follows: (Banea-Mayambu et al. 1997) AAA-diet, 2.5 mg/kg body weight (bw) NaCN; (Berg et al. 2013) AAA-diet, 50 mg/kg bw NaOCN; (Boivin et al. 2013) AAA-diet, equivalent amount of vehicle (1 μl/g bw saline); (Chabwine et al. 2011) SAA-deficient diet, 2.5 mg/kg bw NaCN; (Cliff et al. 2011) SAA-deficient diet, 50 mg/kg bw NaOCN; (Crist et al. 1973) SAA-deficient diet, equivalent amount of vehicle (1 μl/g bw saline). Dose selection for cyanide was informed by previous findings of sublethal and/or lethal cyanide poisoning prior to konzo outbreaks (Banea-Mayambu et al. 1997). Cyanate was given at doses similar to doses known to induce neuropathy in humans (Ohnishi et al. 1975).

### Specific protocols

#### Animal observations

Rodents were examined daily in an open field for physical signs including walking difficulties, tremors and hind limb extension reflexes, which were elicited when the animal was gently raised by the tail. Motor function was assessed by animal performance on an accelerating rotating rod. Animals were individually placed on rotating rods in a software-driven rotarod apparatus (AccuScan Instruments, Inc., Columbus, OH) set in an accelerating mode. The rotation speed was gradually increased from 5 to 25 r.p.m. The apparatus had an automatic system for fall detection *via* photobeams. Rotarod performance was analyzed to estimate how odds of remaining on the rod for at least 60 seconds changed over time. Testing was carried out only in animals maintained on SAA diet to determine whether SAA dietary restriction had an impact on the toxicity of cyanide, our main experimental paradigm of interest.

#### Tissue preparation for proteomics

Serum samples were obtained on day 14 and 28 of the study period. Blood was collected from the salphenous vein using vacutainer tubes with no anticoagulants from all rats and kept overnight at 4°C. The serum was then collected in sterile tubes and centrifuged at 15,000 rpm for 15 min at 4°C. Serum was aliquoted in cryotubes and stored at -80°C till later LC-MS/MS studies. At study termination, rats were deeply anesthetized with 4% isofluorane (1 l oxygen/min) and transcardially perfused with saline through the ascending aorta to remove the remaining blood. The spinal cords were dissected out, flash-frozen in liquid nitrogen, and stored at -80°C. Prior to LC-MS/MS studies, the frozen spinal cord tissue was thawed by adding 500 μl of 100 mM ammonium bicarbonate, and the tissue was uniformly dispersed by 3X15 sec of sonication (Sonic Dismembranator, Model 60, Fisher Scientific). Samples were then centrifuged at 12,000 g for 30 min at 4°C, the supernatant removed, the pellet re-suspended and centrifugated, and the two supernatants pooled. The pellet was then re-suspended in 250 μl 100 mM ammonium bicarbonate by 5 sec of sonication as before. The protein content of the spinal cord soluble fraction, suspended pellet, and serum samples were then determined using the BCA assay (Pierce Chemical, Rockford, IL). The equivalent of 50 μg portions of the two spinal cord fractions and serum were then dried by vacuum centrifugation in preparation for trypsin digestion.

#### Protein digestion

Fifty μg portions of dried serum, soluble and insoluble spinal cord fractions were dissolved in 50 μl of 100 mM ammonium bicarbonate containing 1 mg/ml of Rapigest detergent (Waters Corp., Milford, MA). Eight μl of 50 mM dithioerythritol (DTT) solution was then added and the samples incubated for 60 min at 60º C, followed by addition of 8 μl of 150 mM iodoacetamide solution and incubation at room temperature for 30 min. An additional 16 μl of 50 mM DTT was then added, samples incubated at room temperature for an additional 15 min, and 20 μl of 0.1 μg/μl proteomics grade trypsin added (Sigma Chemical Co., St Louis, MO). Following an overnight incubation at 37°C, samples were acidified by addition of 0.5% trifluoroacetic acid (final concentration), incubation at 37°C for 45 min, centrifugation at 13,000 g for 10 min, and the supernatant frozen prior to analysis of peptides by mass spectrometry.

#### Mass spectrometric analysis

Ten μg samples of peptide digests were injected onto a trap cartridge (Michrom Bioresources, Auburn, CA) at 20 μl/min, samples were washed for 5 min with 2% acetonitrile, 0.1% formic acid, and the trap cartridge placed in-line with a 0.5 × 250 mm SB-C18 reverse phase column (Agilent Technologies, Santa Clara, CA). Peptides were then eluted using a 200 min gradient of 7-30% acetonitrile containing 0.1% formic acid at a flow rate of 10 μl/min. Peptide m/z values (MS spectra) and fragment ions (MS/MS spectra) were collected using an LTQ linear ion trap mass spectrometer (Thermo Scientific, San Jose, CA) with an ion max electrospray source fitted with a 34Ga metal needle. Each survey MS spectrum from 400–2000 m/z was followed by 3 data-dependant MS/MS spectra on the 3 most intense ions found in each survey MS spectrum. The instrument used the dynamic exclusion feature of its control software to ignore previously analyzed ions (repeat count of 1, maximum exclusion list of 50, and 30 sec exclusion time), a tune file configured with one μscan, a maximum ion accumulation time of 200 m/sec, and AGC targets of 30,000 and 10,000, respectively, for MS and MS/MS spectra.

MS/MS spectra from each analysis were converted to dta files using Bioworks 3.3 (Thermo Scientific) and each MS/MS spectrum matched to peptide sequences in a database using the program Sequest (Version 27, rev. 12, Thermo Scientific). Searches were performed with a static modification of +57 on cysteines for carboxyimidomethylation, and differential modifications of +16 for oxidation of methionines, and +43 for carbamoylation of lysines. The search also used trypsin specificity. A database of rat sequences was created from the Uniprot database (Swiss Bioinformatics Institute, Geneva, Switzerland) downloaded in Feb. 2011 (15,524 entries), the sequences of common contaminants added, and the database amended with sequence reversed entries to estimate peptide false discovery rates. Sequest results were then processed using the program Scaffold (version 4.0.5, Proteome Software, Portland, OR) to produce a list of identified peptides and their carbamoylation sites. Peptide and protein confidence levels of 95% and 99% were used, and a minimum of 2 unique peptides matching each protein entry was required.

**Figure 2:**
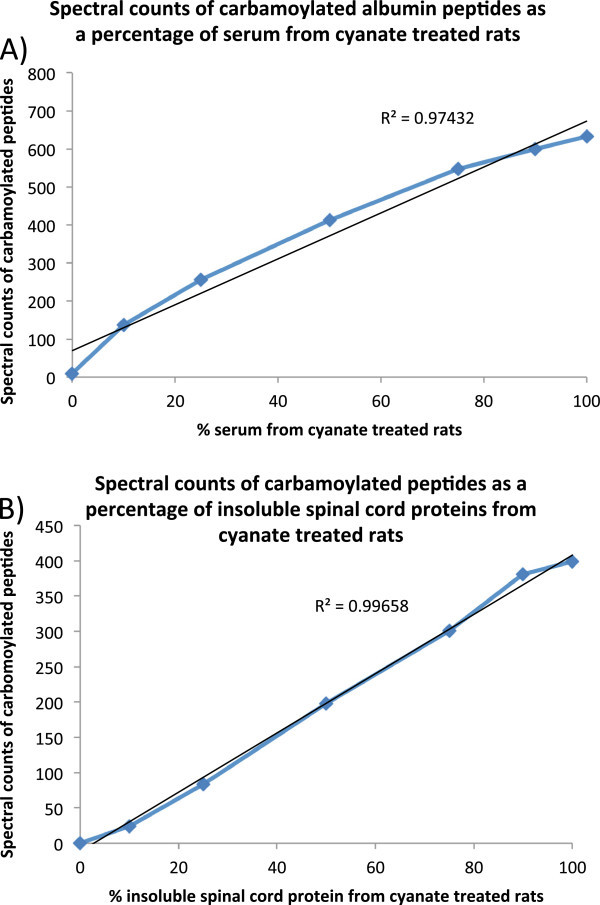
**Tryptic digests from serum (A) and insoluble spinal cord proteins (B) from cyanate-treated and vehicle-only treated rats were mixed and analyzed by LC-MS/MS using an inclusion list for the carbamoylated peptides.** The resulting numbers of MS/MS scans assigned to carbamoylated peptides (spectral counts) were plotted as a function of the % serum and insoluble spinal cord protein from cyanate-treated rats to determine the linearity of the carbamoylation assay for serum albumin and insoluble spinal cord proteins.

Since few carbamoylated peptides were detected in the soluble fraction of spinal cord, further analysis to test for differences in the level of carbamoylation between different treatment groups was only performed for serum and spinal cord insoluble fractions. Differences in levels of carbamoylation in various treatment groups was determined by tabulating the numbers of MS/MS spectra assigned to carbamoylated peptides from albumin in serum digests, and the proteins myelin basic protein, neurofilament light polypeptide, 2’3’-cyclic-nucleotide 3’-phosphodiesterase, myelin proteolipid protein, and glial fibrillary acidic protein in digests of water-insoluble proteins from spinal cord. Inclusion lists for each of the identified carbamolyated peptides was created by specifying the m/z value for each in the instrument’s control software. This maximized the collection of MS/MS spectra for carbamoylated peptides during the quantitative analysis so that a shorter data collection period was required. This mass spectrometric analysis was identical to those described above. However they used a 60 min gradient, inclusion lists specifying the m/z values of the targeted carbamoylated peptides, and the dynamic exclusion feature of the instrument’s control software was disabled. Differences in the level of carbamoylation in various sample groups was then calculated by summing the numbers of MS/MS scans (spectral counts) assigned to carbamoylated peptides from the target proteins in each sample. The MS/MS scans collected during the analysis were again searched using Sequest using the same database and differential searches for modified peptides as before. However, due to the size of the result files, the Sequest results were filtered using PAW software (Wilmarth et al. 2009), which controlled peptide false discovery using numbers of matches to the sequence reversed entries. Peptides identified as being carbamoylated were separately filtered from unmodified peptides to maintain their false discovery rate below 5%. The linearity of the carbamoylation detection was tested by mixing protein digests from cyanate and vehicle treated animals from the sulfur deficient diet group (Figure 2). The numbers of identified carbamoylated peptides as a function of percent cyanate treated serum or spinal cord protein was determined following Sequest searches and filtering of MS/MS data using Scaffold as described above.

### Statistical analysis

The rotorod performance was analyzed using generalized estimating equations (GEE) to estimate the odds of remaining on the accelerating rotating rod for at least 60s changed over time (per each additional day of treatment). At study termination, a secondary analysis of the performance on the rotorod was conducted. An overall (log-rank) test on the time needed to fall off the rotorod among the three groups was carried out. Numbers of carbamoylated sites per protein were assumed to follow a Poisson distribution with the mean number of sites dependent upon diet, treatment, or the interaction between the two. GEE was again used to account for the duplicate samples collected within each animal. Statistical significance was set at 0.05 (0.10 for interaction effects); all p-values are two-sided and confidence intervals set to 95% coverage.
